# Cloning, Expression and Purification of the Beta Subunit of Cholera Toxin Using Escherichia coli as an Expression Host and pET-24a(+) as a Cloning Vector

**DOI:** 10.7759/cureus.101040

**Published:** 2026-01-07

**Authors:** Rudresh M Shoorashetty, Shwetha J Venugopal, Sneha K Chunchanur, Pooja P, Jayapriya R, Himabindu KS, Banani Chakraborty, Ambica Rangaiah

**Affiliations:** 1 Department of Microbiology, All India Institute of Medical Sciences (AIIMS) Bibinagar, Bibinagar, IND; 2 Department of Microbiology, Bangalore Medical College and Research Institute, Bengaluru, IND; 3 Department of Microbiology, Employee's State Insurance Corporation Medical College (ESICMC) and Post Graduate Institute of Medical Science and Research (PGIMSR), Bengaluru, IND; 4 Centre for Nanoscience and Engineering, India Institute of Science, Bengaluru, IND; 5 Department of Microbiology, Bangalore Medical College and Research Institute, Bangalore, IND

**Keywords:** cloning, ctxb gene, pet-24a(+), recombinant cholera toxin b subunit, vibrio cholerae

## Abstract

Cholera toxin has a biologically active A subunit and a binding B subunit. Being non-toxigenic, the Cholera toxin B (CTB) subunit has multiple applications in immunology and rapid diagnostics. A recombinant form of CTB (rCTB) is available for commercial use. However, the COVID-19 pandemic led to a severe global shortage in the production and distribution of commercial CTB, which posed a significant problem for researchers worldwide. Due to the non-availability of commercial rCTB post the COVID-19 pandemic, we attempted and successfully produced rCTB in a pET-based expression system in *Escherichia coli*. To the best of our knowledge, production of rCTB in a pET-based expression system in *E. coli* is reported for the first time in India.

The *ctx*B gene was amplified from toxigenic *Vibrio cholerae* and cloned into the pET-24a(+) plasmid and inserted into *E. coli* DH5α. The plasmid was extracted, and the cloned gene from the pET-24a(+) vector was amplified and Sanger sequenced. The recombinant plasmid was transformed into *E. coli* BL21(DE3) and induced at different temperatures and concentrations of IPTG. The optimum time, temperature, and IPTG concentration were identified, and the rCTB protein was expressed in large culture and purified using Ni-NTA chromatography and dialysed. The protein was sequenced and confirmed as rCTB using LC-MS.

Specific primers designed for the CTB gene amplified a product with the expected size of 341 bp. DNA sequencing confirmed the identity and correct orientation of CTB in the construct and translated using the Expasy translate tool. Optimum conditions for induction were 1mM IPTG, 16℃ for 16 hours. Confirmation of protein was done by liquid chromatography-mass spectrometry. The protein matched with cholera enterotoxin subunit B of *V. cholerae* serotype O1 (strain ATCC 39315) with a score of 82, and the induced protein was confirmed as rCTB. Multiple independent inductions yielded a total protein of ~1+/- 0.1mg per liter of induced culture. The *E. coli *pET-24a(+) plasmid-based expression system is a practical and efficient method for producing rCTB.

## Introduction

Cholera toxin (CT) is a hexameric enterotoxin composed of a single A subunit and five B subunits [1]. Sub-unit ‘A’ is biologically active, while ‘B’ in its pentameric form binds to the GM1 receptor on most nucleated cells [2-4]. Cholera toxin B (CTB) subunit consists of five identical polypeptide chains (each weighing 11.6 kDa), which are non-covalently linked and form a ring-like stable pentameric structure [1,2,4,5].

CTB is non-toxic and has a wide variety of applications [1,2,5]. It was first used as a subunit vaccine for cholera [1]. When chemically or genetically linked to weak immunogens, CTB is a potent mucosal adjuvant, triggering the production of serum and secretory antibodies against the combined antigens [4,5]. CTB has also found application as a transmucosal carrier delivery system for inducing oral tolerance to autoantigens and allergens that have been conjugated [4,5]. CTB has been shown to have potent anti-inflammatory and immunomodulatory effects [5]. Also, it has been found to be a useful target in diagnostic platforms [6].

CTB is commercially available in recombinant form (rCTB). However, due to the COVID-19 pandemic, there has been an acute global shortage in the manufacturing and supply of commercial CTB post-pandemic, a challenge faced by many researchers worldwide. rCTB can be synthesized using bacterial, plant, yeast, and insect expression systems [7,8]. *Escherichia coli* is a valuable organism for high-level expression of heterologous proteins, as it is inexpensive to culture, and expression of recombinant proteins is comparatively fast. To the best of our knowledge, for the first time in India, we have successfully produced rCTB in a pET-based expression system in *E. coli*.

## Technical report

Process

Vibrio cholerae Culture and DNA

A well-characterized clinical isolate of V. cholerae (GenBank Accession number PRJEB50614) was grown in tryptic soy broth at 37°C for 24 hours without shaking. Genomic DNA was extracted from this culture using the Qiagen Mini DNA extraction kit following the manufacturer’s instructions.

ctxB Gene Amplification

The coding sequence for the cholera toxin B subunit gene was downloaded from NCBI (GenBank Accession number MN912820). The sequence coding for mature CTB protein lacking leader peptide (first 21 amino acids from Thr22 to Asn124) was identified (see the Appendix). Specific primers for the ctxB gene were designed incorporating NdeI and NotI restriction sites in forward and reverse primers, respectively (Table 1).

**Table 1:** The primers used for cloning the ctxB gene and T7 universal primers.

Primer sequence (5’ – 3’)		Reference
Primers for ctxB gene		This study
Forward	GGAATTCCATATGACACCTCAAAATATTACTG	
Reverse	ATAAGAATGCGGCCGCATTTGCCATACTAATTGCGGCAATC	
T7 universal primers		Sabirzhanov [9]
Forward	TAATACGACTCACTATAGGG	
Reverse	GCTAGTTATTGCTCAGCGG	

The primers were synthesized by Barcode BioSciences Pvt Ltd, Bengaluru. The PCR test was done in 25 µl of the reaction mixture containing 12.5 µl of Taq DNA PCR Master mix Red (AURA Biotechnologies Private Limited, Chennai), 0.5µl of each primer (10 picomols), 3 µl of extracted DNA, and 8.5µl of nuclease-free water. The PCR test was carried out in duplicate, in the Bio-Rad C1000 touch PCR thermal cycler (Bio-Rad, USA) using the following conditions: denaturation at 95℃ for 5 min, followed by 40 cycles of denaturation at 95℃ for 1 min, annealing at 58℃ for 1 min, and extension at 72℃ for 1 min followed by final extension at 72℃ for 10 min. 

Cloning in the pET-24a(+) Expression Vector

The amplified products were run on 1% agarose gel, and the 341bp band was cut and purified using a Macherey-Nagel gel purification kit. The purified ctxB gene and the pET24a(+) vector were digested with NdeI, followed by NotI restriction enzymes (Takara, Japan) and purified using a PCR purification kit (Macherey Nagel, Germany). The fragments were ligated using T4 DNA ligase (Takara, Japan). The ligated plasmid was transformed into competent Escherichia coli DH5α. The transformants were selected on Luria Bertani agar (HiMedia, Mumbai) with 50µg/ml kanamycin (HiMedia, Mumbai). Ten isolated colonies were inoculated in separate 10 ml LB broth, incubated in a shaker incubator at 37℃ overnight, and the plasmid was extracted from all the clones using a Genei kit and stored for further use. The recombinant plasmid was confirmed for the ctxB gene by restriction digestion & T7 PCR. Positive clones were further verified by Sanger sequencing in both forward and reverse directions (Barcode BioSciences Pvt Ltd, Bengaluru).

Expression of Recombinant CTB Protein

The recombinant plasmid pET24a(+)_ctxB+ was transformed into E. coli BL-21 (DE3) competent cells. The transformed cells were selected on LB agar with 50 µg/ml kanamycin. A single colony was picked up and grown overnight in 10 ml LB broth, and the next day, 1ml of starter culture was added to 100 ml of LB broth with 50µg/ml kanamycin and incubated in a shaker incubator at 37℃ till it reached an OD620 of 0.6. To choose the best conditions for expression, 10 ml of secondary culture was added with 0.5 & 1mM IPTG (HiMedia, Mumbai) and incubated at 4℃, 16℃, and 37℃. After 16 hours, cells were harvested by centrifugation and boiled with sodium dodecyl sulfate-polyacrylamide gel electrophoresis (SDS-PAGE) gel loading dye for five minutes. The total cellular extracts were analyzed in 15% SDS-PAGE. For expression in larger volumes, the best conditions chosen were induction at 1mM concentration of IPTG at a temperature of 16℃ for 16 hours.

Purification of Recombinant CTB

The induction was done in a one-liter volume with 1mM IPTG and incubated in a shaker incubator at 16℃ for 16-18 hours. The cells were harvested, and the pellets were used for protein purification. The 6x His-tag at the C-terminal of recombinant CTB protein facilitates the purification using Ni-NTA agarose beads (Qiagen, USA). For each 1gm of cell pellet, 10ml of the ice-cold lysis buffer (50mM tris-Cl pH 8, 300mM NaCl, 10% glycerol, 1mM PMSF, and 10mM imidazole) was added. The cells are resuspended and then sonicated (pulse on 2s, pulse off 3s at 30% amplitude) for 15 minutes. The lysate was centrifuged at 12000g for 30 minutes at 4℃, and the supernatant was collected; 200µl of Ni-NTA agarose beads (Qiagen, USA) were pre-equilibrated with lysis buffer. The beads were added to the supernatant and incubated with gentle rocking at 4℃ for four hours, and the flow-through was collected. The beads were washed twice with 10-bed volumes of wash buffer A (50mM tris-Cl pH 8, 300mM NaCl, 10% glycerol, and 20mM imidazole) followed by two washes with 5-bed volumes of wash buffer B (50mM tris-Cl pH 8, 500mM NaCl, 10% glycerol and 40mM imidazole). The protein was eluted from the beads by incubation with 2000µl of elution buffer (50mM tris-Cl pH 8, 300mM NaCl, 10% glycerol, and 500mM imidazole). The eluted fractions were collected and checked for purity on 15% SDS PAGE, followed by Coomassie brilliant blue G-250 staining (HiMedia, Mumbai). The purest fractions were pooled and dialyzed against the storage buffer (50 mM tris-Cl pH 8, 50 mM NaCl, 1 mM DTT, and 50% glycerol). The protein concentration was determined using the Bradford method with bovine serum albumin as the standard and stored at -20℃ for further use.

Confirmation of Protein by LC-MS

The purified protein was confirmed by liquid chromatography-mass spectrometry (LC-MS) at the MS facility, Indian Institute of Science, Bengaluru. Briefly, the protein sample was first reduced with DTT and then alkylated using iodoacetamide. This was followed by adding trypsin and incubating at 37°C overnight. Subsequently, the peptides were passed through an Agilent C18 column with a linear gradient from 5% to 95% over 60 minutes at a 0.2 mL/min flow rate. Acetonitrile and water with 0.1% formic acid were used as the mobile phase. LC-MS/MS data were analyzed using the Mascot database.

Results

ctxB Gene PCR & Cloning

**Figure 1:**
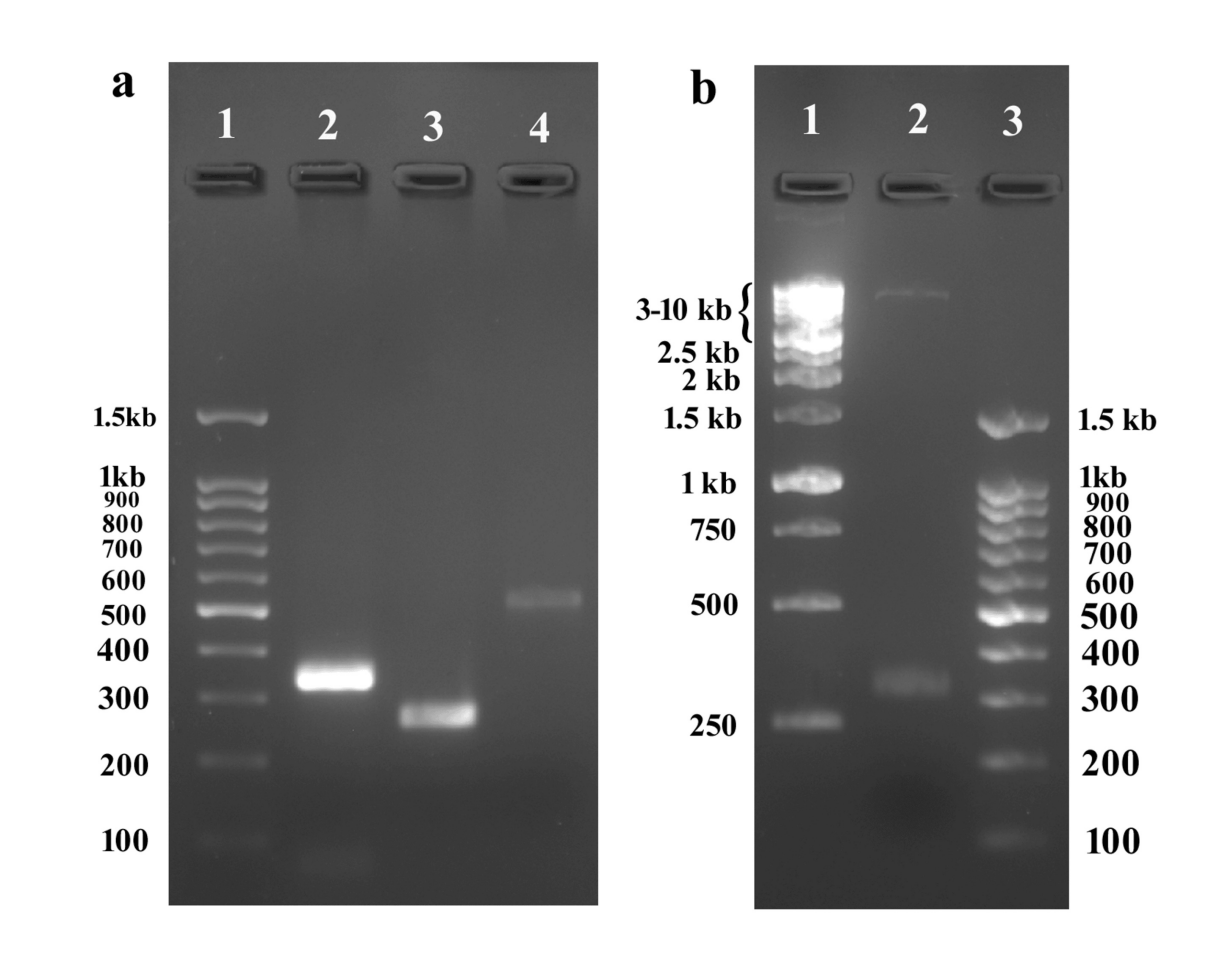
(a) Amplified gene. Lanes: 1, 100bp DNA size marker; 2, amplified ctxB gene (341bp) with restriction sites; 3, T7 PCR using pET24a(+) plasmid; 4, T7 PCR using pET24a(+)_ctxB+. (b) The restriction digestion of pET24a(+)_ctxB+. Lanes: 1, 1kb DNA size marker; 2, digested plasmid showing gene and linear plasmid; 3, 100bp DNA size marker.

The specific primers designed for the CTB gene amplified a product with an expected size of 341 bp. The gene was inserted into the pET24a (+) plasmid and transformed into *E. coli* DH5α and selected on LB agar with 50µg/ml kanamycin. Out of several colonies, 10 colonies were selected, and a recombinant plasmid was extracted. The presence of the gene in the vector was confirmed by restriction digestion and T7 PCR. The restriction digestion with NdeI and NotI showed two bands corresponding to the digested plasmid and the ctxB gene. A 505bp amplicon obtained with T7 PCR indicates that the target gene entered the plasmid correctly (Figure 1).

Finally, DNA sequencing confirmed the identity and correct orientation of CTB in the construct (GenBank accession number PP784253). The DNA sequences were translated using the Expasy translate tool to check for the protein sequence [10], and using the NCBI BlastP tool [11], the output amino acid sequence was searched in the database (see the Appendix). Search results matched to synthetic constructs of the CTB protein.

Expression of Recombinant CTB Protein

**Figure 2:**
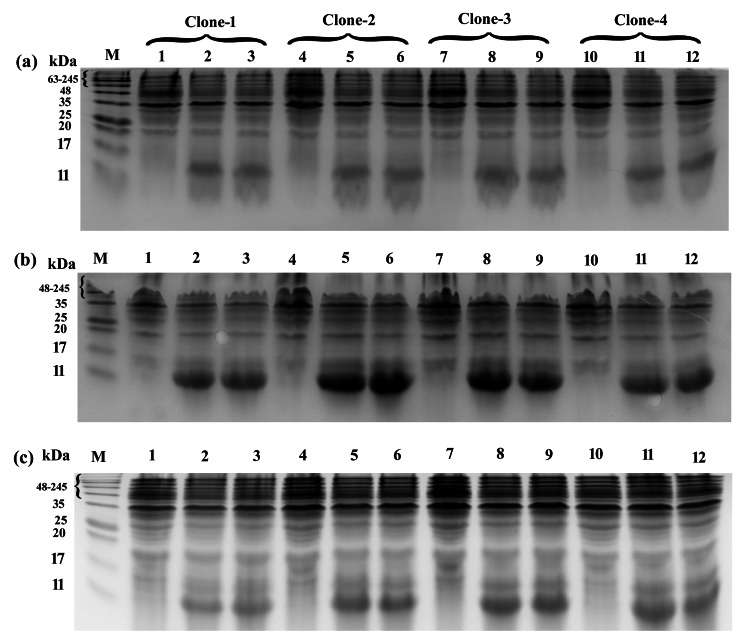
SDS-PAGE analysis of recombinant CTB induction among selected four clones of E. coli BL-21 (DE3) with different IPTG concentrations and different temperatures. M, Protein size marker. (a) Induction at 4℃, (b) Induction at 16℃, and (c) Induction at 37℃. Clone-1 Lanes: 1 uninduced; 2, induction at 0.5mM; 3, induction at 1mM. Clone-2 Lanes: 4 uninduced; 5, induction at 0.5mM; 6, induction at 1mM. Clone-3 Lanes: 7 uninduced; 8, induction at 0.5mM; 9, induction at 1mM. Clone-4 Lanes: 10 uninduced; 11, induction at 0.5mM; 12, induction at 1mM. SDS-PAGE: Sodium Dodecyl Sulfate-Polyacrylamide Gel Electrophoresis

For expression, recombinant plasmid pET24a(+)_ctxB+ was transformed into competent* E. coli *BL-21 (DE3) cells. The total cellular extracts of the induced cells under different IPTG concentrations and incubation temperatures were analyzed in 15% SDS-PAGE (Figure 2).

Good expression was found at 1mM concentration of IPTG at a temperature of 16℃ for 16 hours. The SDS-PAGE analysis showed a single thick band of recombinant CTB with an expected molecular weight of approximately 11kDa.

Purification of Recombinant CTB

**Figure 3:**
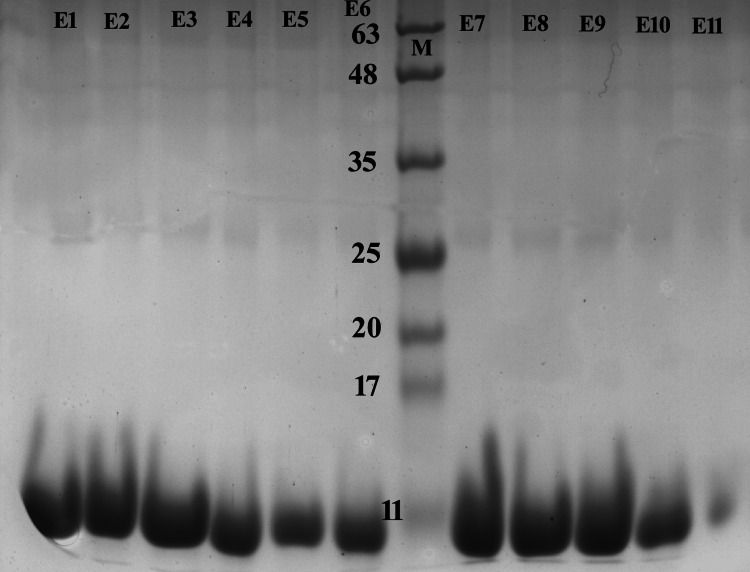
SDS-PAGE analysis of Ni-NTA purified protein elutes E1 to E11. M, Protein size marker. SDS-PAGE: Sodium Dodecyl Sulfate-Polyacrylamide Gel Electrophoresis

The induction was done in a one-liter volume. The CTB protein expressed in the pET24a(+) vector had a 6XHis tag at the C-terminus. This facilitated the purification of the recombinant protein by affinity chromatography through Ni-NTA agarose beads. After washing with buffers A and B, the protein was eluted with 500mM imidazole. The eluted fractions were analyzed using 15% SDS PAGE (Figure 3).

A single band of 11kDa protein was observed. The pure fractions were dialyzed to remove imidazole and high salt content. The Bradford test estimated the amount of rCTB to be about 1mg per litre of induced culture. We observed that qualitatively, a significant portion of expressed rCTB was accumulated in inclusion bodies. Purification was not attempted under denaturing conditions as sufficient protein was purified under native conditions.

Confirmation of Protein by LC-MS

The LC-MS data file was searched in the MASCOT search engine tool using MS/MS Ions search [12]. The protein matched with the cholera enterotoxin subunit B of Vibrio cholerae serotype O1 (strain ATCC 39315) with a score of 82, indicating a significant level of identification as per the MASCOT score (see the Appendix).

## Discussion

CTB’s wide-ranging applications, such as a diagnostic target for rapid platform development, vaccine immunogen/adjuvant, immunomodulator, etc, have made it a popular target. Non-recombinant CTB obtained from *V. cholerae *cultures contains small amounts of whole toxin and its A subunit, which have toxic effects [1]. Though CTB is commercially available, given its drawbacks, it has paved the way for developing rCTB in various expression systems.

Expressing rCTB in *E. coli* is a promising approach. In this study, we expressed rCTB in the *E. coli *pET24a (+) plasmid-based expression system. Plasmid with 6X His-tag at the C-terminal helps effective purification of recombinant protein by affinity chromatography using Ni-NTA agarose, as NTA, which has four chelating sites for nickel ions, binds nickel more tightly than metal-chelating purification systems and provides greater binding capacity and yield. Dertzbaugh et al. demonstrated the affinity of cholera toxin to Ni2+ ions without needing his-tag [13]. However, Okuno et al. demonstrated that the yield of rCTB without His-tag was 20 times less than that of His-tag [14]. Hence, in the present study, the authors decided to introduce 6XHis-tag to CTB to increase yield and to get pure protein.

The full-length ctxB gene (375bp) codes for 124 amino acids, and the first 21 amino acids (leader peptide) are cleaved during post-translational modification to produce mature CTB monomer [8]. The mature monomeric form moves to the periplasmic space and covalently links to form a pentameric structure. The pentameric form is biologically active, essential for CTB function, and acts as a ligand for the GM1 ganglioside receptor. The introduction of mutations and conjugation of heterologous peptides at the N or C terminus will severely affect the production of the pentameric form [8]. Also, altering CTB can significantly hinder its release from host organisms. An alternative to its expression as a secreted protein is extraction from inclusion bodies [1,8]. In the present study, the NotI restriction site was chosen to avoid adding long chains of extra amino acids at the C-terminal of the protein. Although a significant portion of expressed rCTB was accumulated in inclusion bodies, sufficient protein (~1mg per liter of induced culture) was obtained by purification under native conditions. Hence, we didn’t attempt to purify the protein under denatured conditions.

Various studies have used different expression systems, including bacteria, yeast, and plants. Prokaryotes such as genetically modified *V. cholerae*, *E. coli*, Lactobacillus, and eukaryotes ranging from yeast to multicellular organisms such as silkworms and tobacco plants are used to express rCTB [14-18]. However, expression of CTB protein in insects and plants is limited by low yield [2,19] and rCTB has been extensively studied in secretory expression systems, including those utilizing V. cholerae and E. coli [2,4,5,20].

The yield of rCTB in different studies ranges from 1 mg to 50 mg per liter of induced culture, depending on vectors, expression conditions and methods of purification. Owing to this heterogeneity, comparison of yields is difficult. Arêas et al. used a synthetic construct of the ctxB gene and expression in BL21 (SI) strain with NaCl induction [21]. But the expression of proteins using *E. coli* BL21 (SI) strain was discontinued. Boustanshenas et al. used pAE and pQE expression vectors to produce recombinant CTB in *E. coli* BL21 (DE3) cells [22]. pAE plasmid could express a high level of recombinant protein in the fifth hour of induction at 1mM concentration of IPTG [22]. Bakshi et al. introduced point mutation in the ctxB sequence at position 128 (serine to threonine), resulting in overexpression of rCTB, increased solubility, and high yield [19].

Our results revealed that the expression of rCTB in the *E. coli *pET24a(+)_ctxB+ plasmid-based expression system led to good expression of rCTB in native conditions. Purifying the protein in denatured conditions will significantly increase the yield, and introducing PCR-based mutations could improve the expression levels. The *E. coli* expression system is technically simple, inexpensive, and easy to scale up for mass production.

## Conclusions

Versatile applications of CTB coupled with limitations of its commercially available forms have led to the development of rCTB in various expression systems. The need for high yield of rCTB using an inexpensive system made us explore the *E. coli *pET24a(+)_ctxB+ plasmid-based expression system.

We could successfully clone rCTB in the *E. coli *pET24a(+)_ctxB+ plasmid-based expression system. This system induced good expression of rCTB in native conditions. Furthermore, for future applications, purifying the protein in denatured conditions may potentially increase the yield. The *E. coli* expression system, a technically simple, inexpensive, and easy-to-scale-up tool, can be very useful for mass production of rCTB known for its versatile applications.

## Figures and Tables

**Figure 4:**
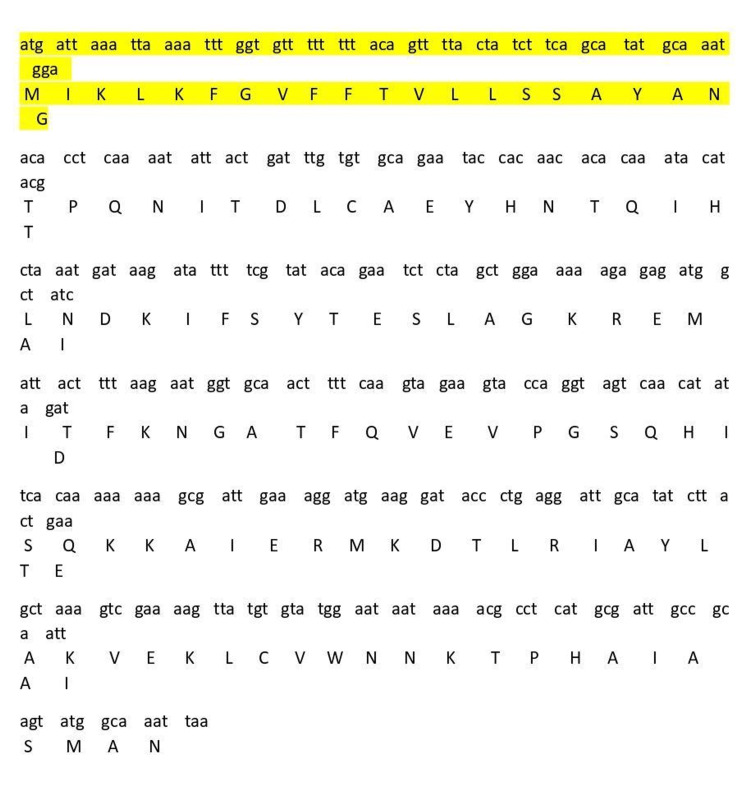
Vibrio cholerae strain—cholera toxin B subunit (ctxB) gene. Complete CDs were downloaded from NCBI (Accession number MN912820) and translated using the Expasy Translate Tool. The highlighted text shows the sequence of the N terminal leader peptide. The sequences coding for leader peptide was removed, and the NdeI restriction site was added to the forward primer and EcoRI to reverse primer.

**Figure 5:**
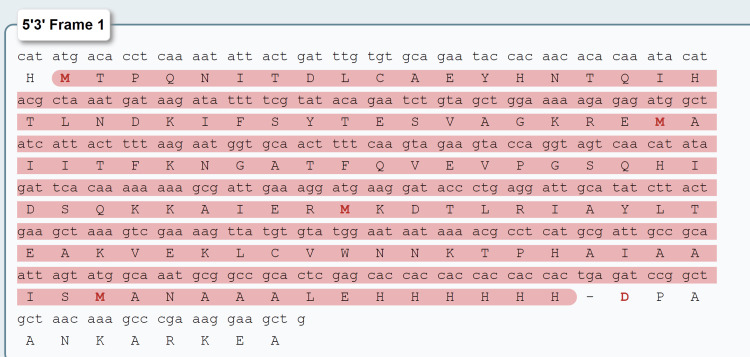
The bidirectional sequencing of the recombinant plasmid using T7 primers, and the sequences obtained were translated using the Expasy translation tool. The highlighted portion represents the rCTB amino acid sequence.

**Figure 6:**
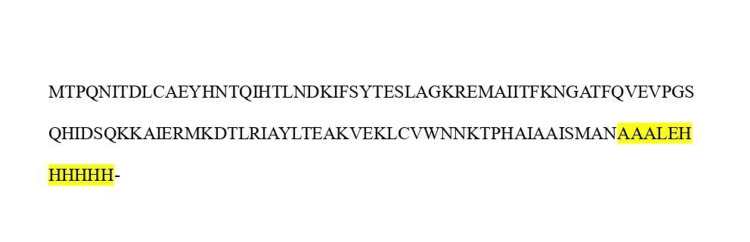
The final amino acid sequence of the rCTB with 6x His tag at the C-terminal end of the protein.

**Figure 7:**
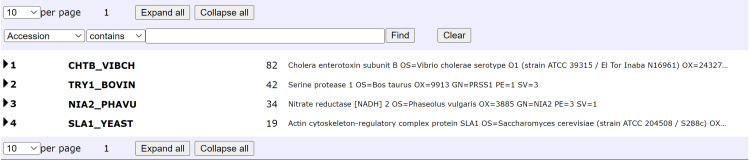
The LC-MS data file was searched in the Mascot SwissProt database. The results matched the Cholera enterotoxin subunit B protein with a score of 82. Three unique sequences were matched specifically with CTB protein. LC-MS: Liquid-chromatography-mass spectrometry
